# Association of hypernatremia with outcomes of COVID-19 patients: A systematic review and meta-analysis: Erratum

**DOI:** 10.1097/MD.0000000000032723

**Published:** 2023-01-20

In the article, “Association of hypernatremia with outcomes of COVID-19 patients: A protocol for systematic review and meta-analysis,”^1^ which published in Volume 101, Issue 36 of *Medicine*, the title has been updated from “Association of hypernatremia with outcomes of COVID-19 patients: A protocol for systematic review and meta-analysis” to “Association of hypernatremia with outcomes of COVID-19 patients: A systematic review and meta-analysis.” The affiliation for Vikash Jaiswal has been corrected from “*Larkin Community Hospital, South Miami, FL” to “*JCCR Cardiology, Varanasi, India.”

